# Antimicrobial Stewardship: A Correct Management to Reduce Sepsis in NICU Settings

**DOI:** 10.3390/antibiotics13060520

**Published:** 2024-06-03

**Authors:** Veronica Notarbartolo, Bintu Ayla Badiane, Vincenzo Insinga, Mario Giuffrè

**Affiliations:** 1 Neonatology and Neonatal Intensive Care Unit, University Hospital “Paolo Giaccone”, 90127 Palermo, Italy; vincenzo.insinga@policlinico.pa.it; 2 Department of Health Promotion, Mother and Child Care, Internal Medicine and Medical Specialties, University of Palermo, 90127 Palermo, Italy; bintuayla.badiane@community.unipa.it (B.A.B.); mario.giuffre@unipa.it (M.G.)

**Keywords:** antibiotics, antimicrobial stewardship, antimicrobial resistance, newborns, neonatal intensive care units (NICUs), antimicrobial stewardship programs, multidrug-resistant microorganisms (MDROs)

## Abstract

The discovery of antimicrobial drugs has led to a significant increase in survival from infections; however, they are very often prescribed and administered, even when their use is not necessary and appropriate. Newborns are particularly exposed to infections due to the poor effectiveness and the immaturity of their immune systems. For this reason, in Neonatal Intensive Care Units (NICUs), the use of antimicrobial drugs is often decisive and life-saving, and it must be started promptly to ensure its effectiveness in consideration of the possible rapid evolution of the infection towards sepsis. Nevertheless, the misuse of antibiotics in the neonatal period leads not only to an increase in the development and wide spreading of antimicrobial resistance (AMR) but it is also associated with various short-term (e.g., alterations of the microbiota) and long-term (e.g., increased risk of allergic disease and obesity) effects. It appears fundamental to use antibiotics only when strictly necessary; specific decision-making algorithms and electronic calculators can help limit the use of unnecessary antibiotic drugs. The aim of this narrative review is to summarize the right balance between the risks and benefits of antimicrobial therapy in NICUs; for this purpose, specific Antimicrobial Stewardship Programs (ASPs) in neonatal care and the creation of a specific antimicrobial stewardship team are requested.

## 1. Introduction

The discovery and use of antibiotics have certainly reduced the incidence of deaths secondary to infectious events; however, their use is not always based on rational evidence and it is often inappropriate. This last phenomenon leads to an increase in bacteria capable of resisting antibiotics, which, therefore, may render them no longer effective when necessary [1].

Antibiotic resistance is an aspect of the broader concept of antimicrobial resistance (AMR) that also involves antivirals, antifungals, and antiparasitics [2]. Bacteria, viruses, fungi, and parasites, in fact, are able to change their characteristics, adapting to the new conditions in which they find themselves and developing new ways to resist the selective pressure given by antimicrobial drugs. Consequently, every time a new antimicrobial molecule is introduced, its effectiveness is destined to be significantly reduced in a very short period [3]. Antibiotic resistance depends on the ability of microorganisms to acquire resistance genes through phenomena of the exchange of genetic material (i.e., pathogenicity islands, PAIs, [4,5] and plasmids) and on new spontaneous mutations of their genome. In the administration of a combined antimicrobial therapy, the spectrum and duration of the treatment determine a positive selection of resistant bacteria favoring their growth and spread in place of those susceptible to antibiotics [3]. In Europe, recent data have shown that >35,000 deaths are caused by infections from multidrug-resistant microorganisms (MDROs) every year [6]. The Centers for Disease Control (CDC) declared AMR a global threat to public health already in 2013 [7].

### 1.1. Use of Antimicrobial Drugs in NICUs, Short-Term and Long-Term Effects

Newborns are particularly exposed to infections due to the poor effectiveness and the immaturity of their immune systems. For this reason, in Neonatal Intensive Care Units (NICUs), the use of antimicrobial drugs is often decisive and life-saving, and it must be started promptly to ensure its effectiveness in consideration of the possible rapid evolution of the infection towards sepsis [8]. Some neonates, such as very low-birth-weight (VLBW) or extremely low-birth-weight (ELBW) ones, can be more susceptible to infection onset and, although signs and symptoms could be very non-specific, antibiotic therapy is started when suspicion of sepsis arises [9]. Moreover, the neonatologist may be induced to maintain antibiotic therapy even in the presence of negative cultures [9]. The optimal duration of therapy for culture-negative sepsis remains unknown up until now [10], but it is recommended to stop antibiotic therapy after 36–48 h if cultures remain negative and the newborn has no clinical signs of infection. This type of “sparing-approach” could reduce the number of treated neonates from 50% to 7.2% [11]. The massive and inappropriate use of antimicrobial drugs in the neonatal period is not only associated with the development of antibiotic resistance but also with several short-term (e.g., adverse effects of the drug, alterations in microbiota composition) and long-term (e.g., increased risks of allergic disease and obesity in childhood) effects [12]. Antibiotics can alter hepatic and renal function and their use can be associated with an increased risk of comorbidities such as necrotizing enterocolitis (NEC), bronchopulmonary dysplasia (BPD), retinopathy of prematurity (ROP), invasive fungal infections, and mortality in ELBW [13]. On the contrary, prolonged exposure to antibiotics increases the risk of non-communicable diseases (i.e., obesity and immune-related disorders, such as asthma and allergic sensitization) in full-term newborns, maybe secondary to intestinal microbiota changes [14]. The inappropriate use of broad-spectrum antibiotics for a long period can alter the normal commensal bacterial flora, thus favoring the proliferation of opportunistic pathogens with increased risks of morbidity and mortality [15]. The alteration of the microbiota in the early stages of life can modify the response of the immune system with long-term repercussions up to adulthood. In fact, it has been demonstrated that newborn microbiota alterations may persist even after antibiotic cessation [7].

### 1.2. Antimicrobial Stewardship Programs: Perspectives

The use of an electronic medical device with the possibility of an electronic prescription could be used to reduce the number of days of antibiotic therapy [10]. As can be seen from the aforementioned work by McCarthy et al. [10], the use of a computerized drug prescription might be useful, for example, in generating schemes for the automatic suspension of antibiotic therapy after 36–48 h from a negative blood culture. The most important national antimicrobial stewardship (AMS) initiative for short-term care settings is the Centers for Disease Control and Prevention’s Get Smart Campaign. Its goal is to promote timely and appropriate antibiotic usage for hospitalized patients, both for empiric and definitive use or prophylaxis [16].

The aim of this narrative review is, starting from the current knowledge, to find the right balance between the risks and benefits of antimicrobial therapy that could fit well in most NICUs, regardless of the demographic and clinical differences of the working reality, by providing the creation of an AMS team, responsible for choosing a specific therapeutic scheme, as part of Antimicrobial Stewardship Programs (ASPs) that can be “tailored” in neonatal care.

## 2. Materials and Methods

A narrative review was performed according to the most recent available literature (2011–2024). Original papers, clinical trials, meta-analyses, and reviews in English were included. Case reports, series, and letters were excluded. The research was conducted using the following keywords (alone or in combination): children, newborns, preterm, gut microbiota, dysbiosis, antibiotic stewardship, drugs, antimicrobial resistance, multidrug resistant microorganisms, neonatal intensive care unit, antibiotic stewardship programs and antibiotic stewardship team. PubMed and Scopus were used as the electronic databases.

## 3. Antimicrobial Resistance (AMR) and Multidrug-Resistant Microorganisms (MDROs)

Antimicrobial resistance is one of the most serious public health threats today, which has been accelerated by the overuse and misuse of antimicrobials in humans and animals plus inadequate infection prevention [17]. The use of broad-spectrum antibiotics invariably alters the flora of the newborn who is taking them, creating a circumstance whereby resistant bacteria have a selective advantage for growth [18]. The increasing incidence of MDRO infections is a source of concern worldwide, as these are microorganisms capable of undermining the safety of patient care [19]. MDRO infections are more difficult to treat, incur greater treatment costs, and are associated with greater mortality and morbidity (i.e., neurodevelopmental outcomes) than infections caused by organisms susceptible to antibiotics [12,20]. Despite the low quality of evidence, a systematic review conducted by Bueno Silva et al. [21] demonstrated an association between the inadequate use of antimicrobial agents and the increase of resistant healthcare-associated infections (HAIs) in neonatal units. Furthermore, antibiotic misuse and overuse not only facilitate the development of MDROs but they also increase unintended consequences, such as *Clostridioides difficile* infections (CDIs), viral and fungal infections (e.g., invasive candidiasis), and antibiotic-associated adverse drug events (ADEs) [17]. Subtherapeutic or low antibiotic tissue concentrations (all other things being equal) predispose to resistance.

### 3.1. MDROs in NICUs

Antibiotic-resistant bacteria are an increasing problem in NICUs. Ineffective empiric antibiotic therapy is associated with an increased risk for morbidity and mortality, especially because it is associated with MDRO circulation [22]. Examples of MDROs in NICU settings include methicillin-resistant *Staphylococcus aureus* (MRSA), vancomycin-resistant *Enterococcus* (VRE), *Klebsiella pneumoniae* expressing extended-spectrum β-lactamases (ESBLs), and Gram-negative bacilli resistant to third-generation and fourth-generation cephalosporins and/or carbapenem groups [18,23]. Recent studies have shown an increase in nosocomial infections in newborns associated with MDROs, even if the literature data are lacking in this slice of the population [24].

Low gestational age, low birth weight, and the extended duration of hospital stay are the main risk factors for colonization or infection with MDROs [25].

### 3.2. Clostridioides difficile Infections (CDIs)

*Clostridioides difficile,* previously known as *Clostridium difficile*, is a spore-forming, anaerobic, intestinal pathogen. It is widely thought that this microorganism is antibiotic-related, and *Clostridioides difficile* diarrhea (CDD) represents a difficult challenge to solve [26]. Clindamycin and beta-lactams (excluding ceftriaxone) are the most frequent antibiotic causes of CDD. Most other antibiotics have low *C. difficile* potential, for example, macrolides, tetracyclines, aztreonam, aminoglycosides, and trimethoprim-sulfamethoxazole. Some antibiotics are actually protective against *C. difficile*, for example, doxycycline and tigecycline [27]. In selecting an antibiotic, practitioners should consider *C. difficile* potential as well as resistance potential.

## 4. Misuse of Antibiotics and Changes in Neonatal Microbiota

Microbiota is the set of microorganisms that are present in a specific niche in the human body (for example, the gastrointestinal tract). Bacteria, viruses, fungi, parasites, and *archaea* are part of it and the bacterial component is the most represented [28,29,30,31]. Gut microbiota is characterized by different bacterial richness and diversity, which tend to evolve over time. After birth, strict anaerobes (e.g., *Firmicutes*, *Bacteroidota*, and *Actinobacteria*) colonize the gut and, after the initial exposure to human milk, their concentration arises along with a consensual reduction in *Proteobacteria*. At 2–4 years of age, adult-like microbiota occurs, with a relative abundance of *Bacteroidota* and *Firmicutes* [14]. Many factors can influence the composition of children’s gut microbiota and among these is antibiotic exposure early in life [32]. The massive use of broad-spectrum antibiotics can alter and modify microbiota composition and maturation by eradicating beneficial microbes and reducing the diversity of bacterial species and strains, with a consequent “dysbiosis” phenomenon [32,33]. Infants who receive antibiotic therapy in the first phases of life have difficulty in regaining typical commensal microbiota even after breastfeeding; maybe, this is due to a change in the natural developmental trajectory of gut microbiota [34]. A prolonged exposure to antibiotics is associated with a reduction in *Bifidobacteria* and *Bacteroides* and with an increase in *Enterococcus*. In particular, Amoxicillin is associated with *Bifidobacterium adolescentis* reduction [14]. During antibiotic therapy, the majority of *Bifidobacteria* is literally eliminated (not simply reduced). As *Bifidobacteria* are fundamental in promoting gut health, the defense against pathogens, and the digestion of human milk oligosaccharides (HMOs), their removal can be associated with negative potential effects on infant growth and development [34]. The increase in potentially pathogenic bacterial strains (e.g., *Enterobacteriaceae*), after the prolonged use of antibiotics, may precede bloodstream infection in preterm neonates. Moreover, the massive use of antibiotics can promote the colonization of newborns with antimicrobial-resistant Gram-negative bacteria [35].

### 4.1. Comorbidities of Intestinal Dysbiosis and Antibiotic Resistance Genes

Intestinal dysbiosis is associated with a higher incidence of both short-term comorbidities (e.g., BPD, LOS and NEC) and long-term comorbidities (e.g., obesity and allergic disease), as demonstrated by the NICU Antibiotics and Outcomes (NANO) Trial [13]. Bacterial community changes can cause effects on the immune development, metabolism, and growth of the infant. At the same time, subtherapeutic concentrations of antibiotics in early life are associated with altered immunity and carbohydrate metabolism, thereby predisposing the host to adiposity later in life [14]. So, it is fundamental to limit the administration of drugs to when it is strictly necessary and to use the correct dosage [36]. For this purpose, it would be desirable to have specific antibiotic stewardship programs, especially for preterm infants, in order to reduce the short- and long-term consequences resulting from the inappropriate use of antibiotics [37]. The association between Amoxicillin and Cefotaxime is the most detrimental to microbial community composition, microbiota development, and antimicrobial resistance gene profile. On the contrary, Penicillin + Gentamicin alters the composition of microbiota less. Probably, this is due to the minimal penetration of aminoglycosides into the gut lumen when administered intravenously [34]. The presence of antibiotic resistance genes (ARGs), also known as “resistome”, in the infant gut microbiota is a significant problem because it makes the treatment of infections more difficult and, often, inefficient [14]. MDROs are able to carry antibiotic resistance genes, posing a severe threat to global public health and human health [38]. In particular, it has been demonstrated that the presence of ARGs is a predictor of neonatal sepsis and adverse birth outcomes [39].

### 4.2. Changes in Newborns’ Microbiota Due to Antibiotic Therapy during Pregnancy

Changes in newborns’ microbiota have been also described in children whose mothers underwent antibiotic therapy during pregnancy: maternal antibiotic exposure is associated with a reduction in *Prevotella copri*, the predominant *Prevotella* spp. within the human gut [32]. At the same time, antibiotic use during pregnancy has been associated with a reduction in *Bifidobacteria* count and, as demonstrated in a study conducted by Zou et al. [40], with an increase in resistant bacteria in a NICU setting [14]. Preterm and full-term infants, whose mothers underwent *intrapartum* antimicrobial prophylaxis (IAP), have a reduction in the levels of *Actinobacteria* and *Bacteroidota*, along with an increase in *Proteobacteria* and *Firmicutes* [41]. An association has been noted between intrapartum Ampicillin exposure and Ampicillin-resistant *Escherichia coli* infection at birth [35]. The exposure to co-Amoxiclav (a combination of Amoxicillin and Clavulanic Acid) during pregnancy would be associated with a four-fold risk of NEC in the newborn compared to placebo [42], maybe due to an altered colonized gut; moreover, antibiotic therapy during pregnancy increases childhood obesity and asthma [43,44,45]. As demonstrated in a case–control study conducted by Hirsch et al. [46], there would be an association between antibiotic orders and several independent allergies diagnosis later in life. This phenomenon may be due to the metabolic alterations consequent to the early antibiotic use: drugs seem to alter the immune response by shifting Th_1_/Th_2_ (T-helper) balance toward Th_2_-dominant immunity with a consequent predisposition for atopy development. By altering microbiota composition, antibiotics can modify the metabolites produced: for example, they can affect hormone synthesis pathways (i.e., steroids) or bile acids and amino acid metabolism [14]. A study conducted by Miller et al. [47] has also shown a shift in copper metabolism in the gut microbiota of antibiotic-treated mice, with a consequent alteration in the expressed levels of copper transporters. In a recent study conducted by Nogacka et al. [41], it was observed that IAP would be associated with a reduction in fecal acetate levels in infants. Given the important roles that the short-chain fatty acids (SCFAs)—of which acetate is a part—play in human physiology, the reduced concentration of this metabolite during the first months of life could have potential effects on infants’ health [48]. Moreover, it has been demonstrated that newborns whose mothers received IAP had genes in their microbiota conferring resistance to the antibiotics used in IAP (β-lactams) [41]. For this reason, the increasing use of antibiotics in pregnant women is becoming a topic of concern for the future health status of the offspring.

## 5. Antimicrobial Stewardship

Antimicrobial stewardship (AMS) is defined as a set of coordinated interventions designed to improve and measure the appropriate use of antibiotics, selecting the best drug for that condition and “tailoring” the dose, duration of therapy, and route of administration for a specific newborn [9,10,36]. The term’s first appearance was on PubMed in 1996. The term was inspired by a Sunday homily about the gospel of the good steward, contributing to the support of the church. Today, its meaning is associated with the careful and responsible management of antibiotics [36]. The aim of the correct use of antimicrobial drugs is to safeguard the long-term public health objective of ensuring sustainable access to available pharmacological resources [49]. The updated Core Elements for Hospital Antibiotic Stewardship Programs by the CDC [50] establish that it is necessary to dedicate human, financial, and information resources to the Hospital Leadership Commitment. Four strategies are needed:

(1) To prevent infections;
(2) To diagnose and treat infections correctly;
(3) To use antimicrobials responsibly;
(4) To prevent the transmission of MDROs [51].

A study conducted by Nzegwu et al. [52] demonstrated that implementation of a NICU-specific antimicrobial stewardship program is feasible and can improve antibiotic prescribing practices, thus reducing drug utilization and, consequently, the antimicrobial resistance phenomenon. In particular, the key principles applicable in a NICU setting are as follows:

- The identification of newborns who really need antibiotic therapy;
- The knowledge of the microbial epidemiology of the NICU, to initiate the empirical therapy;
- The use of the most “tailored” antimicrobial drug(s) without overlapping antimicrobial activity;
- The use of a correct posology (i.e., the selection of the optimal antimicrobial drug regimen, dose, duration of therapy, and route of administration);
- The change in therapy according to the results of cultures and antibiograms and interrupting therapy when it is not strictly necessary [1,21,53].

After the isolation of a specific microorganism, a “tailored” antibiotic therapy must be started, suspending the unnecessary one [54]. At this point, empirical therapy has to be stopped and the drug with the narrowest activity must be used (de-escalation concept) [55]. In this regard, for example, the last Enhanced Recovery After Surgery (ERAS) Society Recommendations suggest the administration of appropriate preoperative antibiotic prophylaxis within 60 min prior to skin incision and the discontinuation of postoperative antibiotics within 24 h of surgery, unless ongoing treatment is required in neonatal intestinal surgery [56,57,58].

In Table 1, the areas of intervention of AMS in a NICU setting are schematized.

### 5.1. Main Strategies to Reduce AMR in NICUs

#### 5.1.1. Prescriptive and Pre-Authorization Limitations

There are strategies to improve antibiotic use by requiring clinicians to obtain approval for certain antibiotics before they are administered [17]. They are easy to apply but, if not adequately supported, these limitations can be associated with an increase in the use of other unrestricted drugs as observed in a study conducted in a Brazilian tertiary-care hospital [65]. In some centers, the main limitation in the application of these interventions could be the lack of a 24 h continuous service. In this sense, telemedicine and the use of artificial intelligence could help [49]. Moreover, a strategy to limit the prescription of carbapenem antibiotics should be in place and to support this strategy, alternatives to carbapenems should be available (e.g., aztreonam, temocillin, fosfomycin, and tigecycline) [66].

#### 5.1.2. Audit and Feedback (A&F) for Prescribers

A periodic review of drug prescriptions and feedback to the neonatologists are recommended to lead to correct education and lasting change over time [17]. These practices do not interfere with clinicians’ sense of autonomy, although clearly shared perspectives and objectives between clinicians and members of the antimicrobial stewardship team are necessary in order to have adequate results [67].

#### 5.1.3. Guidelines’ Implementation

The use of digital tools (e.g., smartphones and tablets) can be fundamental in improving adherence or guidelines already available and constantly updated. These accessible tools help clinicians and comfort them in the most difficult choices when faced with extremely critical newborns [68], making them a beneficial addition to tackling AMR [69]. Nevertheless, international guidelines must be adapted to the specificities of each NICU and this causes quite a few problems. Moreover, although digital tools seem to have a very positive impact on reducing AMR, some studies have found AMS fatigue post-implementation [70,71], maybe due to an excessive alert, such as drug interactions and allergies [72]. A diminished effect of antimicrobial stewardship programs over time has been demonstrated due to the diminished clinicians’ compliance with the guidelines over time, highlighting the importance of designing interventions that can maintain lasting effectiveness [72].

#### 5.1.4. Training of Healthcare Personnel

Training for prescribing, administering, and monitoring antimicrobials must be supported [66]. In particular, the use of social media can be helpful in disseminating up-to-date educational materials to professionals [73]. The global action plan by the World Health Organization (WHO) tries to optimize the use of antimicrobial agents by stressing the need for awareness, education, and knowledge. This program has also developed the AWaRe (Access, Watch and Reserve) classification of antibiotics as a tool to monitor AMS activities [74]. Training and continuous information alone are not enough and must always be combined with other intervention strategies.

#### 5.1.5. Information Technology

As previously mentioned, electronic medical records can be useful for tracking interventions accurately, with automatic checks and alarms on dosages, the duration of therapy, and perioperative prophylaxis [75]. A real-time interface with laboratories can implement clinical decisions, identifying the correct target population to which antimicrobial therapy should be addressed.

#### 5.1.6. Antimicrobial Cycling

If it is true that combinations of antibiotics do not prevent the emergence of resistance in nosocomial pathogens, antimicrobial cycling, or crop rotation, might be helpful in preventing bacterial resistance. This strategy consists of predetermined changes in the empirical usage of antibiotics that occur according to a prespecified time schedule, with the aim of avoiding the prolonged use of a class of antibiotics and the consequent selection of resistant strains [18,76]. Nevertheless, according to the most recent literature, antimicrobial cycling has more disadvantages than advantages and there is no reason to believe that it could improve antibiotic resistance rates within hospitals; thus, “crop rotation” is not recommended up until now [77].

### 5.2. Antimicrobial Stewardship Programs

Antimicrobial stewardship programs try to reduce the unconditional use of any antimicrobial drug by monitoring antibiotic prescribing patterns and antimicrobial resistance and by reviewing the post-prescription of them. Few studies are present in the literature about ASPs in neonatal care, although antibiotic use in neonates is not only associated with AMR but with potential other risks such as invasive fungal diseases [2,78]. The core components of an ASP differ between institutions but as a minimum should include (1) the monitoring of antibiotic prescribing patterns and antimicrobial consumption in days of therapy (DOTs), (2) AMR surveillance, and (3) post-prescription review [2,49]. ASPs have been shown to improve patient outcomes, to reduce antimicrobial adverse events, and to decrease AMR. There is a synergy between infection prevention and antimicrobial stewardship in reducing AMR [17]. Despite the variations in practices, as illustrated by Lu et al. [23] and others [79], certain fundamental principles of ASPs still hold true across all the NICUs (see Table 2).

Limited evidence is available to determine the most effective ASP strategies in the NICU but general principles should apply [80]. Antibiotic policies and guidelines have been shown to be effective in the NICU [81]. Calculating the overall and type-specific antimicrobial consumption can help in establishing and monitoring the functions of antimicrobial stewardship activities as demonstrated by Balkhy et al. [62]. An indicator that can be used for measuring antimicrobial consumption is the “defined daily dose” (DDD), corresponding to the quantity of antibiotic needed to treat a 70 kg adult. A neonatal DDD has been proposed for the eight most commonly used antimicrobials, considering an average weight of 2 kg, but it has not been validated [12]. The usage of electronic medical records and clinical decision support systems to perform AMS activities seems to be an important avenue for time optimization [82].

### 5.3. Management Schemes for EOS and LOS

Both in early-onset sepsis (EOS) and in late-onset ones (LOS), the problem may be false-negative blood cultures, so obtaining adequate blood cultures (0.5–1 mL) upon symptom onset may reduce the number of culture-negative sepsis cases [8,83]. Restricting the initiation or duration of antibiotics in infants based on delivery characteristics, such as the risk of chorioamnionitis along with the use of biochemical markers, can have a substantial impact on the overall use of antibiotics for EOS evaluations. In fact, prolonged antibiotic therapy (≥5 days) in EOS has been associated with several consequences: NEC, an increased risk of subsequent infections, and death [33]. On the other hand, if LOS is suspected, cultures should be obtained from two separate sites to ensure that the bacterial growth represents a true infection rather than a contaminant [35]. Recent data have shown that a specific algorithm-guided treatment might allow clinicians to reduce the misuse of antimicrobial drugs, as demonstrated in a retrospective, observational study conducted in a neonatal and pediatric population in the USA [84]. A specific algorithm based on multivariate models (i.e., Neonatal Early-Onset Sepsis Calculator) may be used to assess the early-onset Group B streptococcal (GBS) infection risk in infants >35 weeks gestational age [85,86]. A prolonged clinical observation can be another important instrument to be pursued. This tool seems to have reduced the administration of antibiotics by about 50% [33]. Molecular methods for GBS detections are also available. The main advantage of polymerase chain reaction (PCR) is the short time frame necessary to provide results regarding GBS colonization status at the time of delivery. However, due to lower sensitivity, in comparison with cultural enrichment, the latest CDC guidelines for GBS prevention recommend antenatal *Streptococcus agalactiae* screening via nucleic acid amplification tests (NAATs) only after prior cultural enrichment in selective broth. Moreover, it must be noted that even a PCR may not provide results fast enough for the sufficient administration of peripartum antibiotics, since many women deliver within a few hours of hospital admission. A further disadvantage is the lack of information on antibiotic susceptibility, which is required for all women reporting an allergy to penicillin. Therefore, the CDC currently do not recommend routine GBS screening using NAATs at delivery [87].

On the other hand, infants <35 weeks gestational age are at the highest risk of early-onset infection from all causes, including group B streptococci, and they have to be approached using the circumstances of preterm birth to decide management [86]. The diagnosis of early-onset GBS infection is made via blood or cerebrospinal fluid (CSF) culture. Evaluations for late-onset GBS disease should be based on clinical signs of illness in the child. The isolation of group B streptococci from the blood, CSF, or other normally sterile sites is fundamental in achieving the diagnosis. The evaluation for GBS late-onset disease (LOD) should also include urine culture [37].

### 5.4. Antimicrobial Stewardship Team

**Figure 1:**
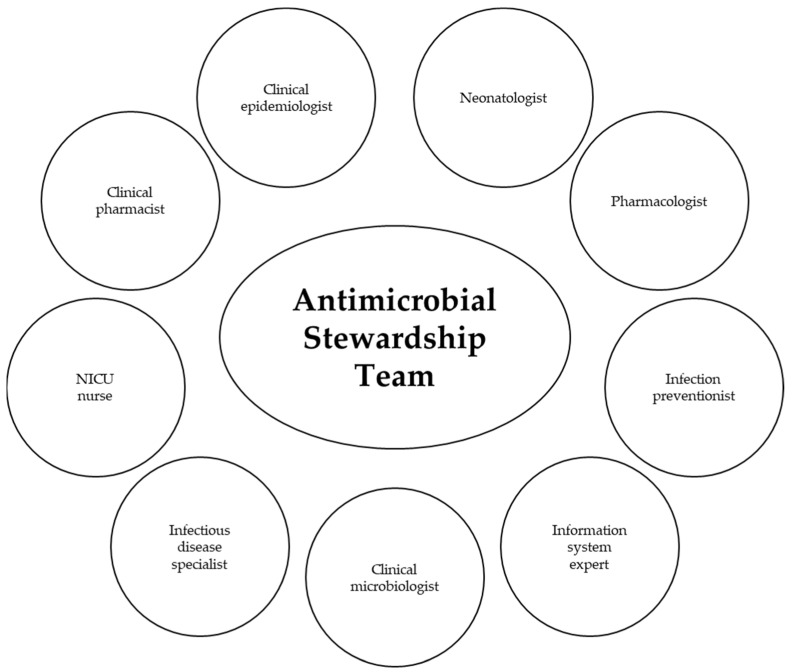
AMS Team.

The neonatologist is the clinician responsible for identifying the health needs of newborns as well as the risks to which they may be exposed, starting the diagnostic process and choosing the appropriate treatment. However, the implementation of an antimicrobial stewardship program in the NICU cannot be achieved by neonatologists alone [88]. It is necessary to establish a team of specialists with different skills (i.e., clinical epidemiologist, pharmacologist, clinical microbiologist) who share the main objective of the responsible use of antimicrobial drugs and who intervene at all possibly interested levels: from the choice of the best strategy to its application, from the monitoring of the process to its ongoing re-evaluation, and from the measurement of the results obtained to the analysis of the risk/benefit ratio [61]. It is fundamental that an antimicrobial stewardship team is present in every healthcare facility, with periodic audits, surveillance strategies, and feedback about the prescription of antibiotics [10]. A leader (i.e., physician) and a co-leader (i.e., clinical pharmacist) have to be responsible for program management and outcomes, by making audits and feedback to improve antibiotic use [89]. It is important to monitor antibiotic prescriptions, to report information on antibiotic use and resistance, and, finally, to educate all the team about adverse reactions from antibiotics and antibiotic resistance [50]. The antimicrobial stewardship team can co-opt additional members depending on the care setting and the antimicrobial issue being considered [59]; see Figure 1. AMS teams in conjunction with clinical specialty and governance teams should be alerted to monitor for the unintended consequences of antimicrobial guidelines and should have robust systems in place to communicate with key clinical personnel regarding antimicrobial prescribing alerts such as issues with resistance or supply [66]. Finally, it is essential to support antimicrobial stewardship teams by developing processes that promote antimicrobial stewardship or by allocating resources to ASP interventions [59,60].

A collaborative approach between several professionals within the team has proven to be much more effective than applying restrictive guidelines developed by experts. The team must identify opportunities and areas of intervention based on the specific characteristics of the NICU to reduce the inappropriate use of antimicrobial drugs [88].

## 6. Antimicrobial Stewardship Programs in NICUs around the World

Over the last 10 years, numerous projects have focused on promoting the responsible use of antibiotics and the evidence published in the international scientific literature has increased exponentially. A collaborative, multicenter quality improvement initiative, conducted by the Vermont Oxford Network, from 2016 to 2017, has shown a reduction in antibiotic use within NICUs during the analyzed period. This result was due to a major adherence and implementation of the CDC’s core elements of antibiotic stewardship (i.e., leadership commitment, accountability, drug expertise, action, tracking, reporting, and education), that mirrored the reduction in unnecessary antibiotic use over the 2-year time period considered [63,64,90].

A recent study conducted in the NICU department of a Japanese community hospital showed that the application of ASPs successfully reduced antimicrobial prescriptions from 175.1 to 41.6 DOTs/1000 patient days, without any significant changes in safety outcomes, such as sepsis, mortality, and treatment failure [91].

The aim of an ongoing prospective study on low-birth-weight neonates (<1500 g) admitted to participating tertiary NICU in Canada is to develop consensus on NICU antimicrobial stewardship interventions to enhance best practices [92]. Using an expanded Canadian Neonatal Network (CNN) platform, physicians will collect data on antimicrobial use and the susceptibility of organisms identified in clinical samples from blood and CSF over a period of 2 years. These data will be used to provide all NICU stakeholders with benchmarked center-adjusted antimicrobial use and MDRO prevalence reports. An ASP plan will be developed at both the individual unit and national levels in the subsequent years [92].

Finally, in a recent prospective, single-center quality improvement project conducted at the American University of Beirut Medical Center (Lebanon), it has been demonstrated that a neonatal-specific antimicrobial stewardship program is associated with a significant and sustained decrease in antimicrobial use (−63% ampicillin use and −79% gentamicin use) [93].

## 7. Conclusions

Antibiotic stewardship is a coordinated program that promotes the appropriate use of antibiotics, improves patient outcomes, reduces microbial resistance, and decreases the spread of infections caused by MDROs. A reasoned and judicious use of antimicrobial drugs in the NICU setting is essential to guarantee an extremely vulnerable population of newborns and can be achieved with multiple coordinated interventions that involve the interaction and collaboration of neonatologists with other professionals from other healthcare specialties. It is of fundamental importance to have therapeutic management protocols that are unique in all NICUs in order to reduce the short- and long-term effects that incorrect management of antimicrobials can cause. The identification of the best antimicrobial stewardship strategies for the neonatal population, especially the most vulnerable, such as extremely preterm newborns or ELBW infants, still requires further studies with long-term follow-up.

## Figures and Tables

**Table 1:** Areas of AMS intervention in NICU setting.

Diagnosis of Neonatal Sepsis	Blood culture is the diagnostic “gold standard” [8]. Use of a sterile procedure to collect the sample (at least 1 mL) [8]. Implementation of clinical tools (i.e., strict clinical follow-up) [59]. Implementation of laboratory investigations (i.e., C-reactive protein, procalcitonin, interleukin-6 and 8 [9,60], rapid molecular assays, and mass spectrometry) [36].
Choice of Empirical Antimicrobial Treatment	Epidemiological and microbiological data of a NICU (surveillance programs) are fundamental in the choice of empiric antibiotic therapy [27]. Routinary empirical antibacterial therapy with very broad-spectrum antibiotics should be avoided or should be discontinued as soon as possible, in order to prevent MDRO infections development [27]. Empiric therapy for early-onset sepsis (EOS), late-onset sepsis (LOS), and NEC must be continually re-evaluated to use the narrowest-spectrum antibiotic therapy [61]. For fungal infections: Amphotericin B, Fluconazole (if it has not been used for prophylaxis), and Echinocandins can be administered [62].
Re-evaluation of treatment already underway	Interruption of antibiotic therapy if culture is negative after 36–48 h of incubation and clinical progress is not suggestive of sepsis [13,60]. After the isolation of a specific microorganism, the most appropriate drugs must be used, suspending the unnecessary ones [55].
Measurement of blood levels of antimicrobial drugs	Adapt the pharmacological dosage according to the gestational age, the days of life, and the presence of any other pathologies of the newborn [55]. When possible, carry out the plasma dosage of the drug to evaluate the correct therapeutic dose range [2].
Perioperative prophylaxis	Single drug (i.e., cefazolin) can be used for prophylaxis for no more than 24 h [63]. Need for standardized operating protocols [37].
Continuous monitoring of the use of antimicrobial drugs with performance indicators	The calculation of days of therapy (DOTs) per 1000 days of hospitalization is an indicator of antimicrobial consumption that can be used for comparing the performance of a NICU to others [64].

**Table 2:** Main principles of ASPs in NICU setting.

Clear documentation of baseline data and dedicated guidelines within a specific AMS group	Necessity of close work between the medical staff and the microbiology laboratory, to control infections [34]. Guideline development should take into account local and national emerging antimicrobial resistance, local and national surveillance of antimicrobial use, local qualitative data on prescribing, and rates of *C. difficile* infections [37]. In fact, although the core elements of successful ASPs are similar between programs, differences between hospital programs are expected and necessary given that each hospital has unique ASPs [34].
Application of appropriate A&F mechanisms	Enthusiastic support from the administration as well as the medical staff [34]. Hospital antimicrobial guidelines should be readily accessible to prescribers and subject to regular review by the Antimicrobial Management Team with a formal review at a minimum of every 3 years [37]. The assessment tool can be used on a periodic basis (i.e., annually) to document current program infrastructure and activities and to help identify items that could improve the effectiveness of the stewardship program (i.e., hospital leadership commitment, accountability, pharmacy expertise) [50].
Avoidance of prolonged empirical antibiotic therapy with automatic discontinuation when clinical and biochemical conditions allow it	Treating infection and not colonization [34]. Preferentially selecting antibiotics with a low potential resistance [34]. Avoiding, if possible, high-resistance-potential antibiotics [34]. In EOS, it appears reasonable to administer 10 days of drugs in uncomplicated bacteremia, until 14 days for GBS meningitis and up to 21 days for Gram-negative organism meningitis [27]. In LOS, 3 to 5 days of antimicrobial therapy could seem a reasonable timing to reduce antibiotic resistance phenomena, when clinical improvement occurs in the newborn 48 h after the start of antibiotic therapy [27].
